# Research on catalytic denitrification by zero-valent iron (Fe^0^) and Pd-Ag catalyst

**DOI:** 10.1371/journal.pone.0266057

**Published:** 2022-04-15

**Authors:** Zhen Jiao, Yu Zhou, Zhijia Miao, Xueyou Wen, Yupan Yun

**Affiliations:** 1 School of Energy and Environmental Engineering, University of Science and Technology Beijing, Beijing, China; 2 School of Water Resources and Environment, Institute of Intelligence and Environment Industry Technology, Hebei Province Collaborative Innovation Center for Sustainable Utilization of Water Resources and Optimization of Industrial Structure, Hebei Province Key Laboratory of Sustained Utilization and Development of Water Resources, Hebei GEO University, Shijiazhuang, Hebei, China; Universiti Brunei Darussalam, BRUNEI DARUSSALAM

## Abstract

This study primarily focused on how to effectively remove nitrate by catalytic denitrification through zero-valent iron (Fe^0^) and Pd-Ag catalyst. Response surface methodology (RSM), instead of the single factor experiments and orthogonal tests, was firstly applied to optimize the condition parameters of the catalytic process. Results indicated that RSM is accurate and feasible for the condition optimization of catalytic denitrification. Better catalytic performance (71.6% N_2_ Selectivity) was obtained under the following conditions: 5.1 pH, 127 min reaction time, 3.2 mass ration (Pd: Ag), and 4.2 g/L Fe^0^, which was higher than the previous study designed by single factor experiments and orthogonal tests, 68.1% and 68.7% of N_2_ Selectivity, respectively. However, under this optimal conditions, N_2_ selectivity showed a mild decrease (69.3%), when the real wastewater was used as influent. Further study revealed that cations (K^+^, Na^+^, Ca^2+^, Mg^2+^, and Al^3+^) and anions (Cl^-^, HCO_3_^-^, and SO_4_^2-^) exist in wastewater could have distinctive influence on N_2_ selectivity. Finally, the reaction mechanism and kinetic model of catalytic denitrification were further studied.

## Introduction

Contamination with nitrate (NO_3_^-^) in water resource has attracted increasing public concern. Nitrate detected in water body is a common contaminant that may cause severe health risks, such as blue baby syndrome, cancer, as well as the eutrophication of water bodies [1]. Agricultural activities (mainly the over-fertilization of nitrogenous fertilizers), atmospheric deposition, and sewage discharges mainly contribute to nitrate pollution [2].

Several technologies have been developed for treatment of nitrate-contaminated water, including physico-chemical denitrification (such as ion exchange, reverse osmosis, chemical precipitation, and electrocoagulation), biological treatment, and chemical reduction [3]. Among these approaches, biological denitrification, and catalytic hydrogenation enable to selectively reduce nitrate to nontoxic nitrogen (N_2_) [4, 5]. However, the biological method requires intensive maintenance, excessive biomass disposal, and constant addition of carbon resources [6]. In recent years, the technology of chemical catalytic reduction of nitrate attracts more attention. In 1989, Vorlop and Tacke first put forward the traditional chemical catalytic hydrogenation that utilized the reductant H_2_ and bimetal catalyst for nitrate reduction [7]. In this catalytic process, catalyst plays the indispensable role, while H_2_ has been regarded as the reductant, which provides the active H that can participate in the deoxidation process of the nitrate reduction. However, the low solubility of H_2_ in aqueous media and the operational complexity (appropriate H_2_ flow rate, pressure) have been the big problem [8]. Several researchers replaced H_2_ with organic acid (e.g., HCOOH) or its salt (e.g., NaCOOH) to convert nitrate to N_2_ [9]. However, the incomplete decomposition of acid or its salt and the threat to human health greatly restricts its wide application. Based on these, the novel synergistic effect of zero-valent iron (Fe^0^) and bimetallic catalyst for nitrate reduction was proposed.

The experimental design for evaluating and optimizing experimental parameters can minimize costs and maximize desired responses [10, 11]. For most researchers, the single factor experiments and orthogonal tests have been widely used for experimental design. However, these two methods are incapable of getting true optimal conditions due to ignoring the interactions among influential variables [12]. Therefore, instead of these two methods, Response Surface Methodology (RSM) was utilized for the optimization of catalytic denitrification conditions in this paper. RSM is a particular set of mathematical and statistical approach that develops for building models, evaluating the effects of variables, and determining the optimal conditions of variables [13]. This method contributes to completing the comprehensive design with a minimum number of experiments, analyzing the interaction between the parameters, and more directly and accurately obtaining the optimal operation parameters [14].

Actually, until now, RSM has not been used as an optimization tool for catalytic reduction of nitrate. Hence, in this research, as a design framework in RSM, Box-Behnken Design (BBD) was used to model and optimize the processes of catalytic denitrification achieved by zero-valent iron (Fe^0^) and Pd-Ag catalyst. Finally, the reaction mechanism of catalytic denitrification was comprehensively illustrated.

## Material and methods

### Materials

The chemical reagents used in this research were: sodium nitrate (NaNO_3_), silver nitrate (AgNO_3_), palladium chloride (PdCl_2_), hydrochloric acid (HCl), iron powder (<0.07 nm, >98%), graphene, SiO_2_, diatomite, kaolin, γ-Al_2_O_3_, and silica gel. The catalyst (Pd-Ag/graphene) can be obtained through the traditional wet impregnation method [15].

### Experimental design

**Fig 1:**
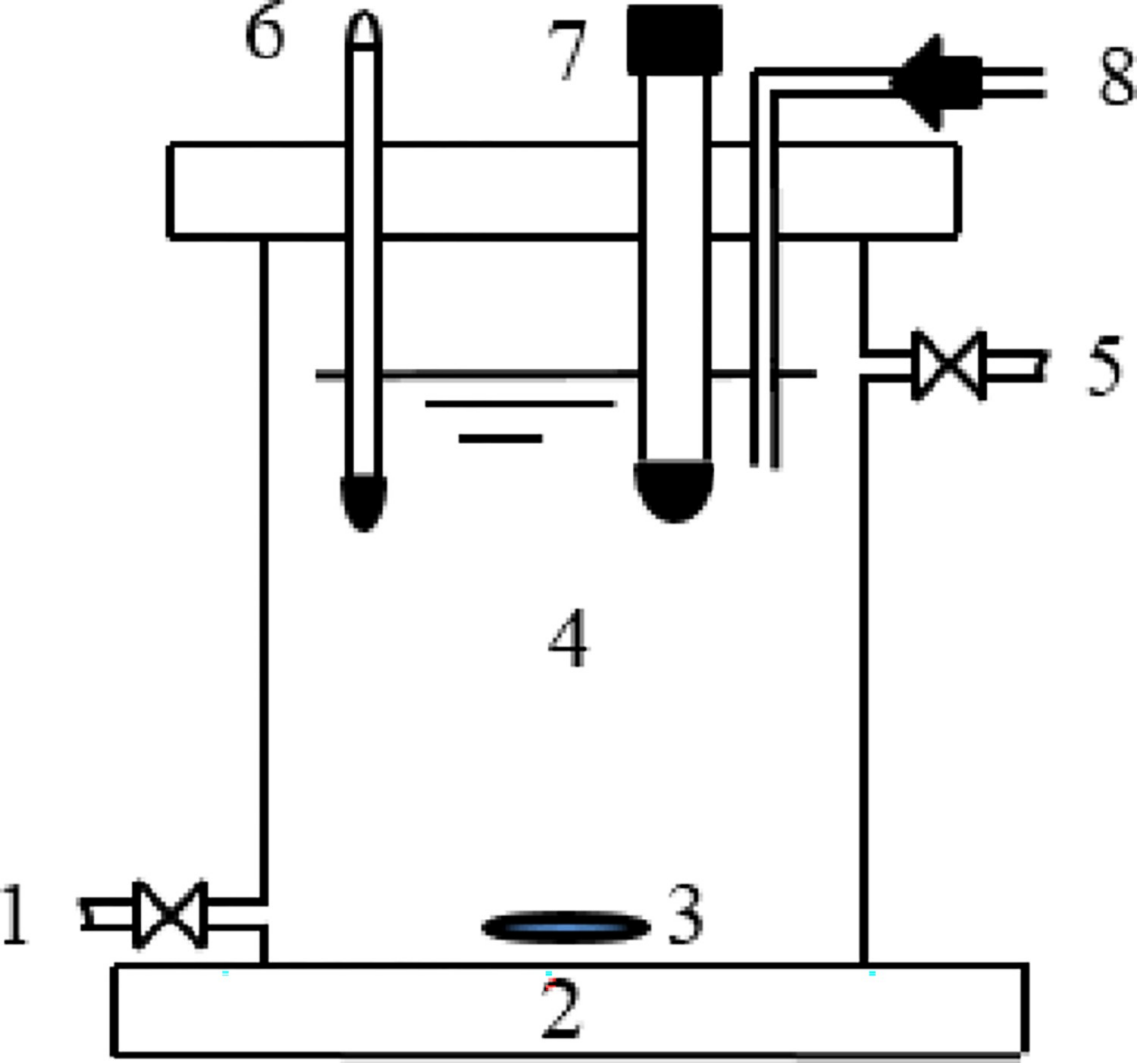
Schematic of the reactor (1: Influent; 2: Magnetic stirrer; 3: Rotor; 4: Reactor; 5: Effluent; 6: Thermometer; 7: pH meter; 8: Automatic titrator).

Batch experiments were completed to investigate the potential factors that may impact catalytic performance. All tests were performed in a 1 L plexiglas reactor (Fig 1). Certain amounts of Fe^0^ and catalysts were added to the reactor prior to the experiments. To guarantee the better mass transfer effect for catalytic denitrification, the reactor was placed on an magnetic stirrer under 450 rpm at room temperature (20±5°C). 1 mol/L HCl was added to reactor by one automatic titrator to remain needed solution pH during catalytic process.

Samples were periodically collected to determine the concentration of nitrate-nitrogen (NO_3_^—^N), nitrite-nitrogen (NO_2_^—^N), ammonium (NH_4_^+^-N) and total nitrogen (TN) after 0.45 μm membrane filtration. NO_3_^-^, NO_2_^-^ and TN were measured with an ion chromatograph (DIONEX-120), while NH_4_^+^ was tested via the Nessler’s reagent spectrophotometry.

The N_2_ selectivity was calculated as:

$$N_{2}\;selectivity\;(\%)=\frac{C_{N_{2}}}{C_{0^{‐}}C_{t}}\times 100\%$$ (1)

Where *C*_*0*_ is the initial nitrate concentration (mg/L), *C*_*t*_ is the nitrate concentration (mg/L) at time t (min), $C_{N_{2}}$ is the content of N_2_ (mg/L).

## Results and discussion

### RSM analysis

(1) Box-Behnken design (BBD)

BBD was used for experimental design. The levels of BBD were shown in Table 1.

**Table 1:** Levels of Box-Behnken design.

Factor	Levels		
	-1	0	+1
**pH(X**_**1**_ **)**	4.1	5.1	6.1
**Time/min ((X**_**2**_ **)**	90	120	150
**Pd:Ag mass ratio(X**_**3**_ **)**	2:1	3:1	4:1
**Fe**^**0**^ **dosage/g/L(X**_**4**_**)**	3	4	5

(2) Regression equation fitting and analysis of variance (ANOVA)

Minitab 19 was applied to the multiple regression fitting. The experiments were conducted and the quadratic multinomial regression equation was listed as follows, and the regression equation coefficients and T test can be seen in Table 2:

Y (N_2_ selectivity) = 69.67 + 0.583 X_1_ + 1.000 X_2_ + 1.833 X_3_ + 1.750 X_4_−17.708 X_1_*X1−2.833 X_2_*X_2_−2.833 X_3_*X_3_−1.958 X_4_*X_4_ + 2.500 X_1_*X_2_ + 1.000 X_1_*X_3_−1.250 X_1_*X_4_ + 0.750 X_2_*X_3_ + 1.250 X_2_*X_4_+ 0.250 X_3_*X_4_

**Table 2:** Regression equation coefficients and T test.

Term	Coefficient	Standard error coefficient	T-Value	P-Value
**Constant**	69.67	1.12	62.14	0.000
**X**_**1**_	0.583	0.561	1.04	0.019
**X**_**2**_	1.000	0.561	1.78	0.100
**X**_**3**_	1.833	0.561	3.27	0.007
**X**_**4**_	1.750	0.561	3.12	0.009
**X**_**1**_ **X**_**1**_	-17.708	0.841	-21.06	0.000
**X**_**1**_ **X**_**2**_	-2.500	0.971	-2.57	0.024
**X**_**1**_ **X**_**3**_	0.500	1.14	0.44	0.670
**X**_**1**_ **X**_**4**_	-1.250	0.971	-1.29	0.222
**X**_**2**_ **X**_**2**_	-2.833	0.841	-3.37	0.006
**X**_**2**_ **X**_**3**_	0.750	0.971	0.77	0.455
**X**_**2**_ **X**_**4**_	1.250	0.971	1.29	0.222
**X**_**3**_ **X**_**3**_	-2.833	0.841	-3.37	0.006
**X**_**3**_ **X**_**4**_	0.250	0.971	0.26	0.801
**X**_**4**_ **X**_**4**_	-1.958	0.841	-2.33	0.038

Note: P < 0.05, significant level; P > 0.05, below significant level [16].

As depicted in Table 2, the linear term- X_1_, X_3_ and X_4_, the interaction terms- X_1_X_2_, X_1_X_4_, and all the square terms- X_1_X_1_, X_2_X_2,_ X_3_X_3_ X_4_X_4_ remarkably affect test results (P < 0.05). Whereas, X_1,_ X_2_, X_1_X_3_, X_1_X_4_, X_2_X_3_, X_2_X_4,_ X_3_X_4_ have no significant impact on the experimental results.

As exhibited in Table 3, P-value = 0.000 <0.01, R^2^ = 90.47, which prove the model built above accurately and the regression equation obtained has been better fitted [17]. Therefore, it comes to the conclusion that this model can be used to continuously analyze and predict experimental data.

**Table 3:** Analysis of variance (ANOVA) results of the quadratic experimental model.

Source	DF	Adj SS	Adj MSS	F-Value	P-Value
Model	14	596.935	42.638	8.13	0.000
Linear	4	138.167	34.542	6.59	0.005
X_1_	1	65.333	65.333	12.46	0.004
X_2_	1	24.083	24.083	4.59	0.053
X_3_	1	36.750	36.750	7.01	0.021
X_4_	1	12.000	12.000	2.29	0.156
Square	4	447.519	111.880	21.34	0.000
X_1_ X_1_	1	436.009	436.009	83.16	0.000
X_2_ X_2_	1	45.370	45.370	8.65	0.012
X_3_X_3_	1	25.037	25.037	4.78	0.049
X_4_X_4_	1	17.120	17.120	3.27	0.096
2-way interaction	6	11.250	1.875	0.36	0.892
X_1_ X_2_	1	1.000	1.000	0.19	0.670
X_1_ X_3_	1	1.000	1.000	0.19	0.670
X_1_ X_4_	1	9.000	9.000	1.72	0.215
X_2_ X_3_	1	0.250	0.250	0.05	0.831
X_2_ X_4_	1	0.000	0.000	0.00	1.000
X_3_X_4_	1	0.000	0.000	0.00	1.000
Error	12	62.917	5.243		
Total	26	659.852			
Lack-of-Fit	10	60.917	6.092	6.09	0.149
Pure error	2	2.000	1.000		

R^2^ = 90.47%.

**Fig 2:**
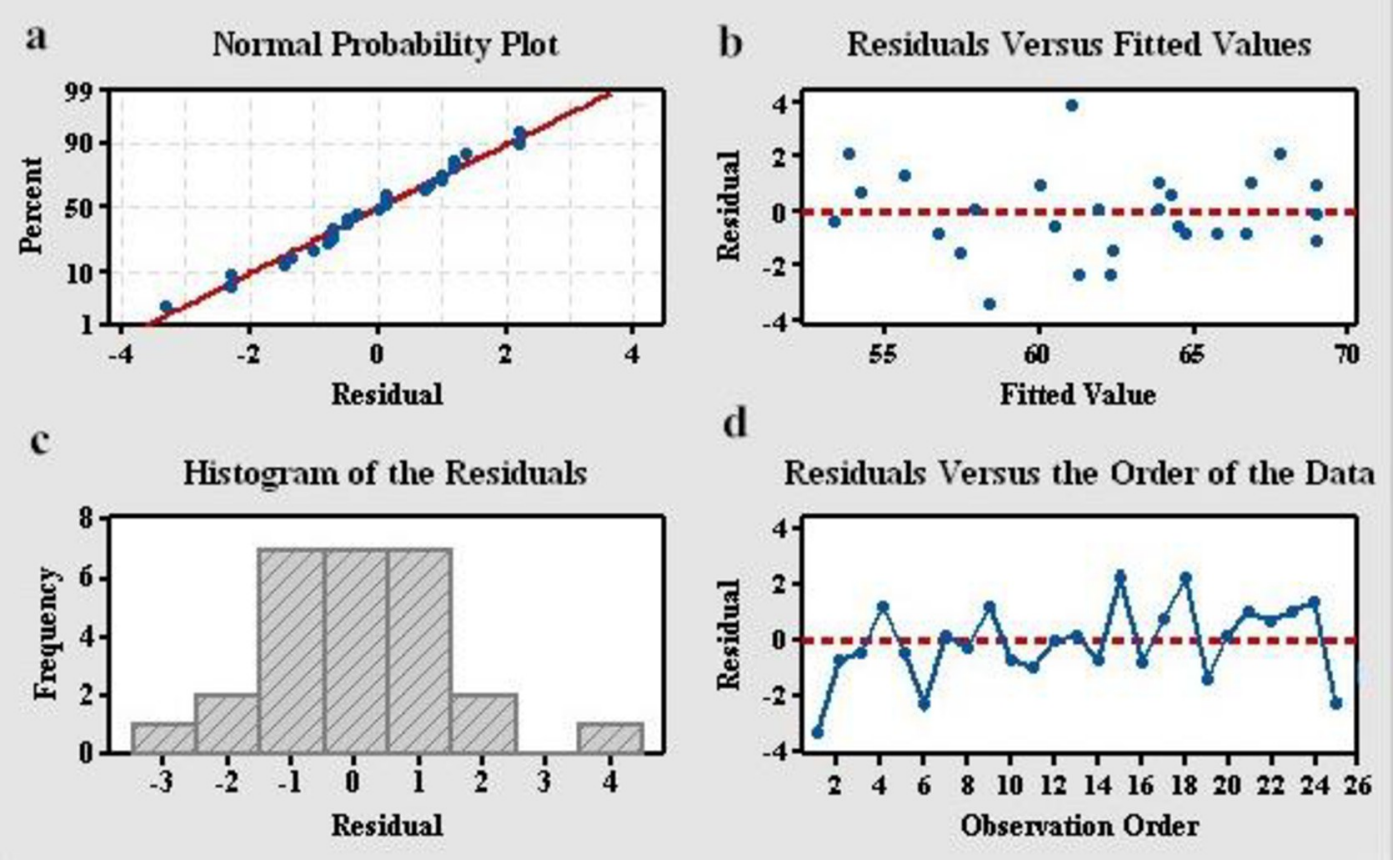
Residual plots for N_2_ selectivity.

In addition, in order to validate the model proposed above, the residual plots were checked, listed in Fig 2. It’s believed that randomness and unpredictability are essential components for any valid regression model. Through the residual plots analyses, whether the observed error (residuals) is consistent with stochastic error can be accurately assessed. The residuals should be centered on zero throughout the range of fitted values indicated in Fig 2B and 2D. Random errors assumed to produce residuals should be normally distributed. In other words, the residuals should fall in a symmetrical pattern and have a constant spread throughout the range which can be proved in Fig 2A and 2C.

(3) 3D response surface analyses

**Fig 3:**
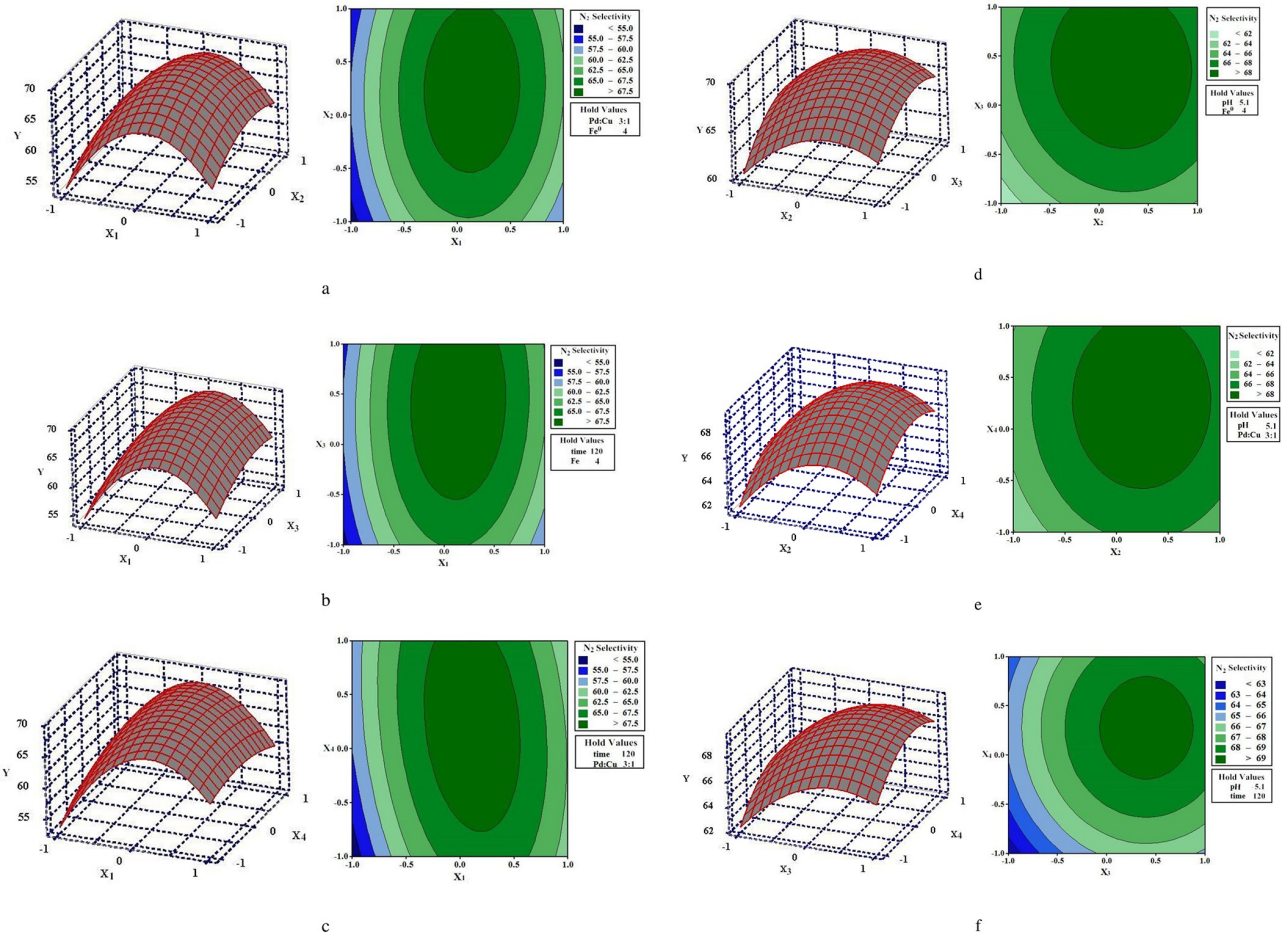
Response surface (Left) and Contour plots (Right) between two factors (a) X_1_ and X_2_; (b) X_1_ and X_3_; (c) X_1_ and X_4_; (d) X_2_ and X_3_; (e) X_2_ and X_4_; (f) X_3_ and X_4_.

3D response surface analyses were further conducted for four factors, including pH, time, Pd:Ag mass ratio, and Fe^0^ dosage, which can be seen in Fig 3. Response surface and contour plots have been applied to intuitively indicate the influence of various factors on N_2_ selectivity, so as to find out the optimal parameters and the interaction between the factors [18]. In the contour plots, the central point of the minimum ellipse is the highest point of the response surface. Additionally, the shape of the contour line can reflect the strength of the interaction, and the oval indicates that the interaction between the two factors is significant, while the circle reflects the opposite meaning.

As depicted in Fig 3A, compare with others, response surface and contour plots of X_1_ and X_2_ on N_2_ selectivity show the significant influence trend, which is consistent with the data in Table 4. In order to obtain the predicted maximum value through the model we build, the canonical analysis of response surface was conducted, which was listed in Table 4.

**Table 4:** Canonical analysis of response surface.

Factor	X_1_	X_2_	X_3_	X_4_	Type of stable point
**Coded value**	0.13	0.23	0.41	0.23	maximum value
**Actual value**	5.1	127	3.2	4.2	69.8%

As indicated in Table 4, the predicted maximum value is 69.8%. The actual values of the four factors (X_1_, X_2_, X_3_, and X_4_) obtained from the coded value are: 5.1 pH, 127 min time, 3.2 Pd: Ag, and 4.2 g/L Fe^0^, respectively, which are the predicted optimal parameters.

(4) Validation test

The validation experiments were conducted under the predicted optimal parameters: 5.1 pH, 127 min reaction time, 3.2 mass ration (Pd: Ag), and 4.2 g/L Fe^0^. Results showed that the N_2_ selectivity of catalytic denitrification reached 71.6%, higher than the study designed by the single factor experiments (68.1%) and orthogonal test (68.7%) in Table 5, which proves that the model used in this research is accurate and can get the true optimal conditions for the catalytic reduction of nitrate.

**Table 5:** N_2_ selectivity with different designs.

Design method	pH	Time (min)	Pd:Ag mass ratio	Fe^0^ dosage (g/L)	N_2_ selectivity (%)
Single-factor design	5.2	120	3:1	4	68.1
Orthogonal test	4.2	120	3:1	5	68.7
RSM design	5.1	127	3.2:1	4.2	71.6

### Simulation experiments of real wastewater

To test the effect of water quality on N_2_ selectivity, real wastewater obtained from the secondary effluent of a municipal wastewater treatment plant in Beijing, China, was adopted for batch experiments. The properties of water samples were: concentration of NO_3_^−^- N: 19.2 mg/L, NO_2_^−^- N: 0.1 mg/L, NH_4_^+^- N: 0.2 mg/L, TN: 21 mg/L, and pH: 6.7. The catalytic conditions were: 5.1 pH, 127 min reaction time, 4 g/L catalyst: Pd-Ag/graphene, Pd:Ag = 3.2:1, Pd: 5 wt%, and 4.2 g/L Fe^0^.

As described in Table 6, compared to the artificial solution (NaNO_3_) as influent, N_2_ selectivity showed a mild decrease as the real wastewater was used as the influent. This phenomenon may be due to the ions that exist in wastewater. Therefore, the effect of the ions on catalytic performance was further investigated.

**Table 6:** Water quality analyses of the effluent.

Water sample	pH	NO_3_^-^-N (mg/L)	NH_4_^+^-N (mg/L)	NO_2_^-^-N (mg/L)	TN (mg/L)	N_2_ selectivity (%)
**Wastewater**	8.4	10.2	3.3	0.2	14.7	69.3
**NaNO**_**3**_	8.2	8.9	3.4	0.1	13.6	71.6

**Fig 4:**
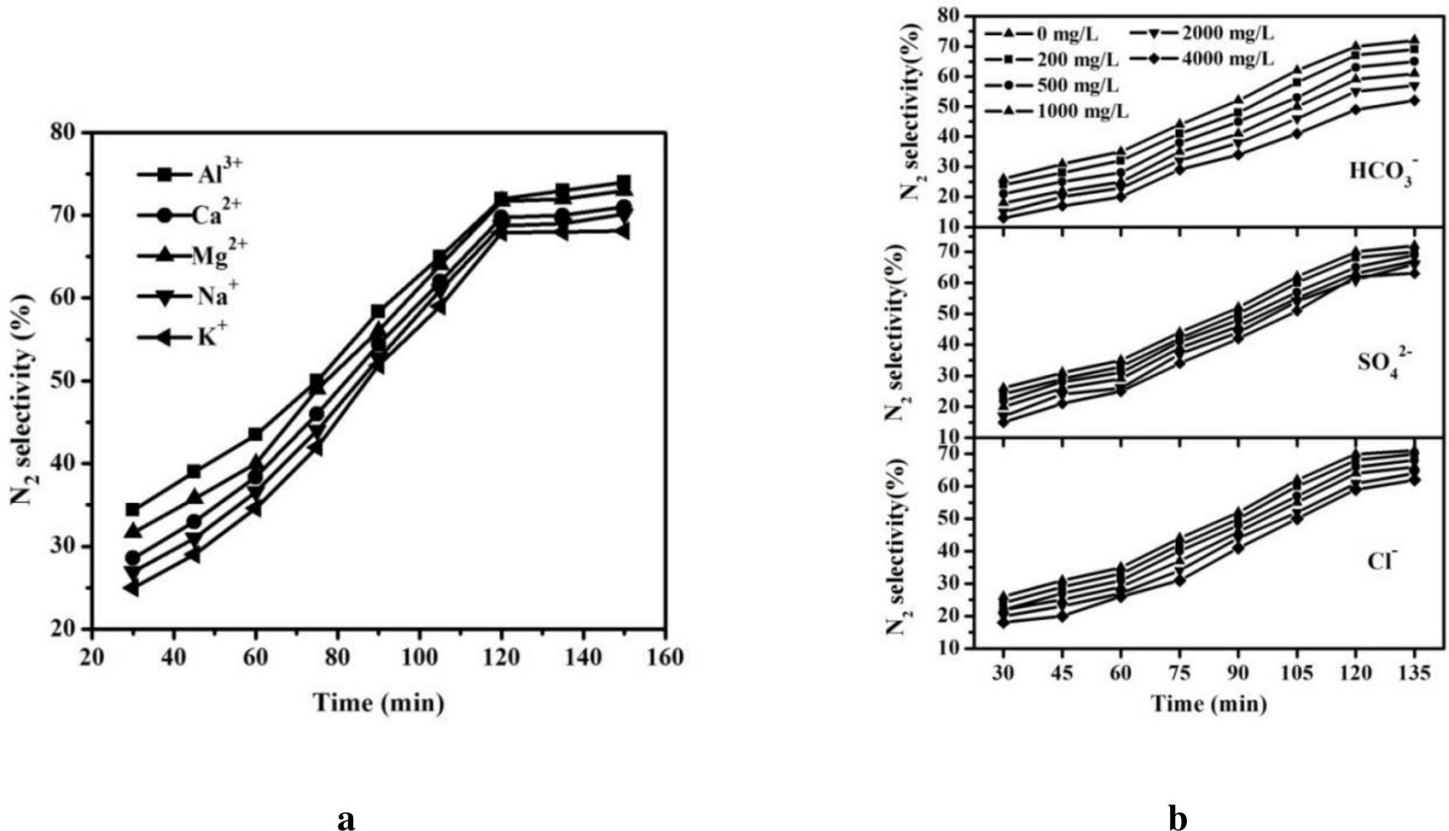
Catalytic performances with different cations (a) and anions (b) in solution.

Fig 4A shows the effect of different cations on N_2_ selectivity for nitrate reduction. 20 mg/L of artificial solutions (Al(NO_3_)_3_, Ca(NO_3_)_2_, Mg(NO_3_)_2_, KNO_3_, NaNO_3_) were prepared prior to the experiment, respectively.

A series of experiments with various nitrate salts as the source of nitrate ions revealed that the catalytic performance increased in the following order: K^+^ < Na^+^ < Ca^2+^ < Mg^2+^ < Al^3+^. It has been reported that these cations have different influence on the migration rate of NO_3_^-^ and OH^-^ in solution [19]. Cations with high valence or small radius seem more likely to have a strong ability to bond with NO_3_-, preventing NO_3_^-^ from catalytic reduction. Similarly, the cations in solution tend to strongly adsorb the formed OH^-^ that may have a negative impact on catalytic denitrification, enhancing the separation of OH^-^ from bimetallic active sites on surface of the catalyst and offering suitable space and conditions for a the catalytic reaction [20].

As depicted in Fig 4B, the impact on N_2_ selectivity with Cl^-^, SO_4_^2-^, and HCO_3_^-^ were respectively investigated. It’s obvious that HCO_3_^-^ partially contributed to the decrease of catalytic performance. The higher the HCO_3_^-^ concentration, the worse the catalytic performance was. This result was mainly derived from the fact that HCO_3_^-^ possesses similar plane structure than NO_3_^-^, leading to the competitive adsorption with NO_3_^-^ on surface of the catalyst, which leads to adverse influence on nitrate reduction [20]. In contrast, due to the different structure, Cl^-^ and SO_4_^2-^ both had little to do with catalytic nitrate reduction [21].

### Reaction mechanism

(1) Role of the reductant-Fe^0^

**Fig 5:**
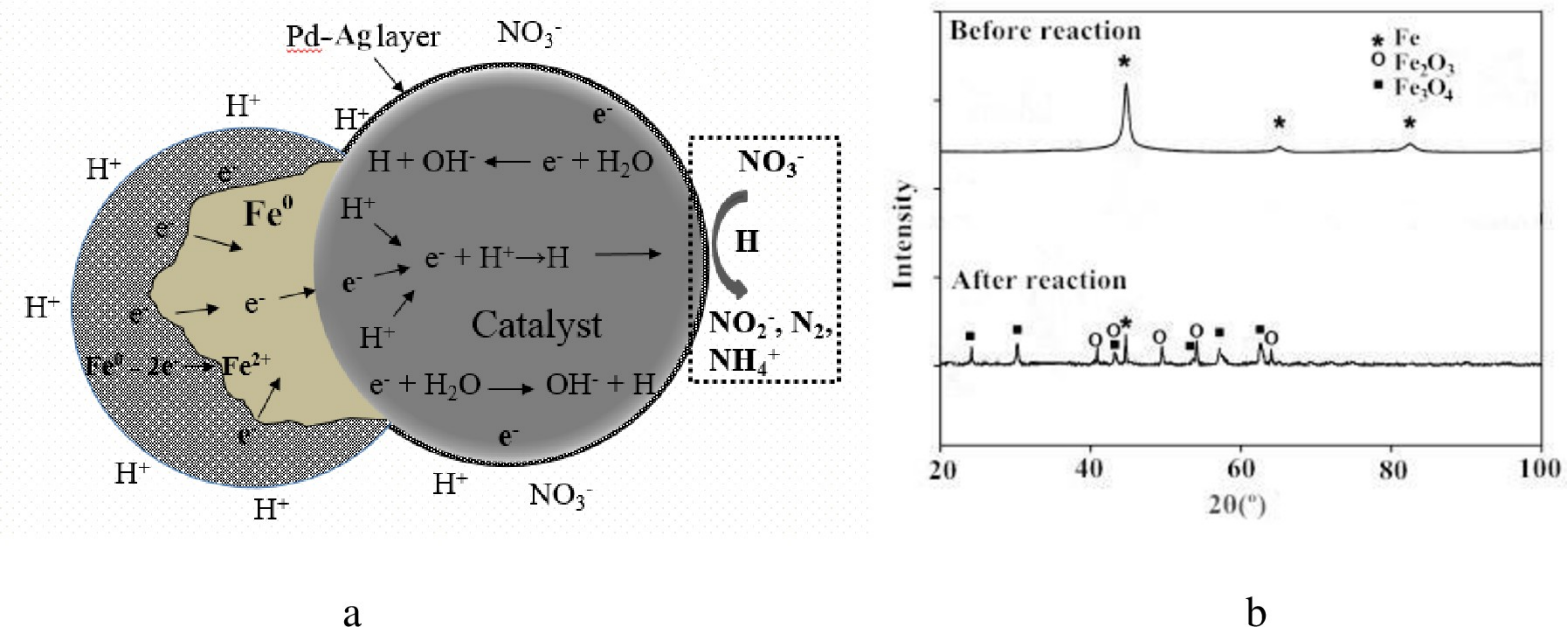
a: Role of Fe^0^ in catalytic process; b: XRD patterns.

Fe^0^ primarily served as electron donor in catalytic process. In general, the catalytic denitrification involved the directional electron transfer from Fe^0^ to nitrate, which is then converted into non-toxic N_2_ or less toxic species (NO_2_^-^ and NH_4_^+^) [22]. In practical terms, at the metal active sites at the surface of carrier, the electron that Fe^0^ lost could bond with H^+^ in solution and form active H, which took part in the deoxidization process and reduced NO_3_^-^, as shown in Fig 5A.

XRD patterns of Fe^0^ before and after catalytic reaction were exhibited in Fig 5B. It’s obvious to find that magnetite (Fe_3_O_4_) and hematite (Fe_2_O_3_) were detected on surface of Fe^0^, which is consistent with the Schlicker’s finding [23]. The possible reaction equations are listed as below:

$$Fe^{0}‐2e^{‐}\to Fe^{2+}$$ (2)

$$2Fe^{0}+3H_{2}O^{}‐6e^{‐}\to Fe_{2}O_{3}+6H^{+}$$ (3)

$$3Fe^{0}+4H_{2}O‐8e^{‐}\to Fe_{3}O_{4}+8H^{+}$$ (4)

(2) Catalytic denitrification process

It’s believed that the catalytic reduction of nitrate has been the stepwise processes. As indicated in Eqs (Eq 5–11) [24], H^+^ receives the electron from Fe^0^, forming the active H, which takes part in the deoxidization process, converting NO_3_^-^ to N species (NO_2_^-^, NH_4_^+^, or N_2_) [25]. It’s worth noting that more N_2_ can be produced, only the appropriate H^+^ concentration in solution has been remained. High H^+^ concentration may lead to the generation of undesired NH_4_^+^, which has to be treated again. Additionally, H^+^ can also reduce the accumulation of OH^-^ generated with the catalytic processes.


$$H^{+}+e^{‐}\to H$$ (5)

$$NO_{3}{}^{‐}+H^{}\to NO_{2}{}^{‐}+OH^{‐}$$ (6)

$$NO_{2}{}^{‐}+H\to NO+OH^{‐}$$ (7)

$$NO^{}+NO^{}\to N_{2}+2O$$ (8)

$$NO^{}+2H^{}\to NH+OH^{‐}$$ (9)

$$NH+NH\to N_{2}+2H$$ (10)

$$NH+2H+H_{2}O\to NH_{4}{}^{+}+OH^{‐}$$ (11)

In the catalytic denitrification processes, catalyst composed of the active ingredients and the carrier significantly influences the catalytic performance [26]. The carrier that supports the active ingredients can provide reaction sites for catalytic reaction [27]. In addition, the physico-chemical properties (pore structure, surface area, mechanical strength, and the chemical components) of the carrier determine the dispersion degree of the supported active metal particles (Pd, Ag) that control the processes of adsorption, diffusion, reaction, and desorption of the reactants (mainly NO_3_^-^, NO_2_^-^) and the products (mainly NH_4_^+^, N_2_) that occurred on the catalyst’s surface, which may greatly affect the catalytic reduction of nitrate [28]. Therefore, the materials that possess the porous structure, larger specific surface area, good adsorptive capacity, and stable physico-chemical properties tend to be selected as the carrier of the catalyst.

**Fig 6:**
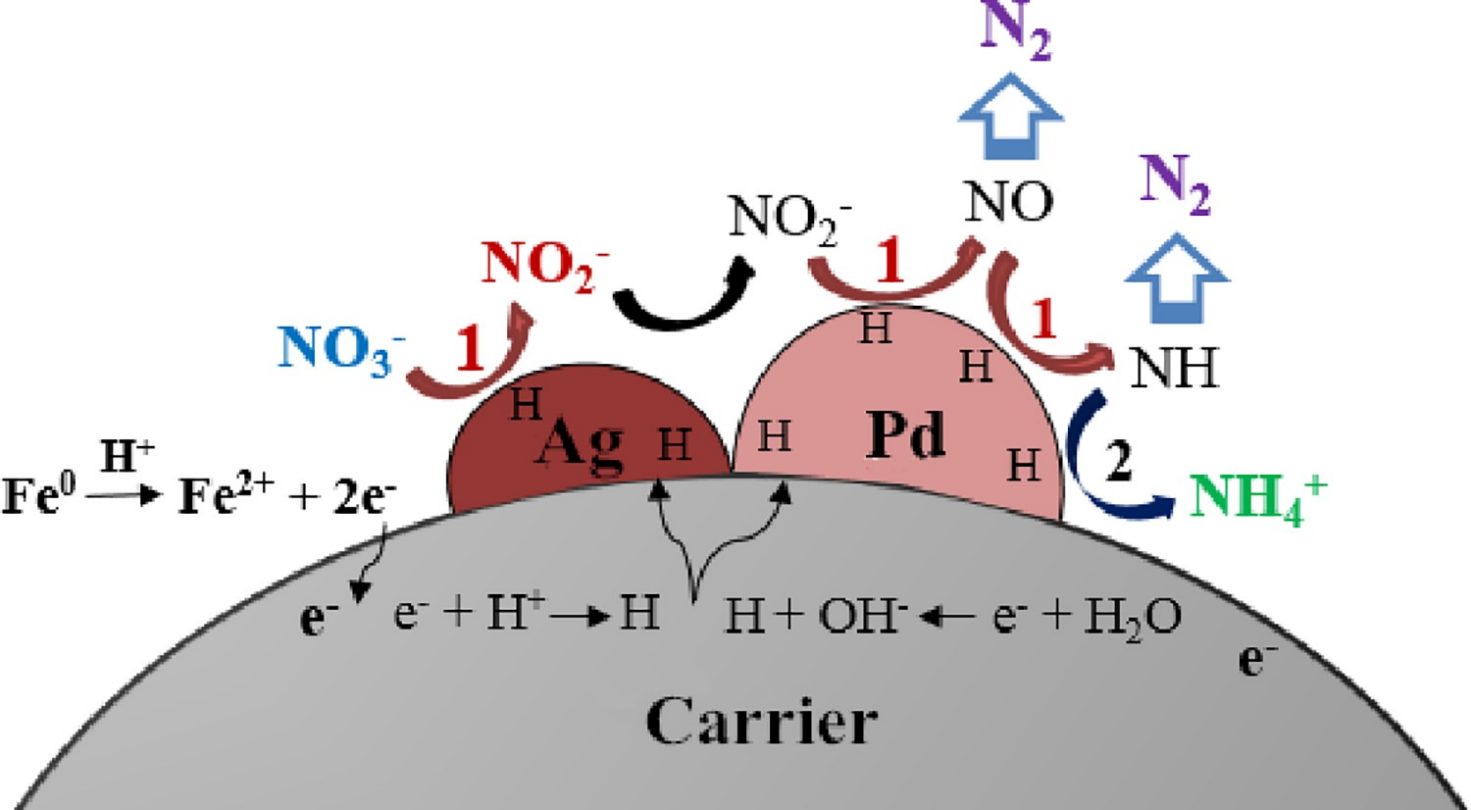
Catalytic process for nitrate reduction.

In addition, the active ingredients can affect the catalytic performance by directly and indirectly participate in the catalytic reaction. Research found that the active ingredients loaded on the carrier should better comprise of a noble metal (such as Pd or Pt) and an auxiliary element (such as Ag, Cu or In) [29]. The bimetallic- Pd and Ag can active the formed H, which involves in the deoxidization process to reduce nitrate. Actually, Ag-H mainly acts with the reactant-NO_3_^-^, producing NO_2_^-^. Furthermore, on Pd active sites, the product- NO_2_^-^ can be continuously reduced to other N species (NO, NH, N_2_, and NH_4_^+^) [30]. The catalytic reaction mechanism is illustrated in Fig 6.

### Kinetic study

Currently, significant research focuses on the kinetics of catalytic hydrogenation. Rare research on the catalytic process using Fe^0^ and bimetallic catalyst to reduce nitrate was conducted. It can be assumed that the zero-order kinetics and first-order equation of Langmuir-Hinshelwood could be employed to describe this process. According to our previous study, the catalytic denitrification process could be better explained by the first order kinetic model [31]. The kinetic equation could be obtained: y = 247.1x +0.1398, R^2^ = 0.9975.

It has been suggested that in the process of catalytic denitrification, the produced intermediates such as NO and NH have been negligible [32]. Based on the first-order equation above, the reaction rates are presented in Eqs 12–15. A kinetic study on catalytic denitrification with different catalysts was further conducted, as listed in Table 7.


$$\frac{dC_{NO_{3}^{-}}}{dt}=-(k_{1}+k_{2}+k_{3})\;C_{NO_{3}^{-}}$$ (12)

$$\frac{{\mathrm{dC}}_{{\mathrm{NO}}_{2}^{-}}}{\mathrm{dt}}{=k_{1}\;C}_{{\mathrm{NO}}_{3}^{-}}{-(k_{4}+k_{5})\;C}_{{\mathrm{NO}}_{2}^{-}}$$ (13)

$$\frac{{\mathrm{dC}}_{N_{2}}}{\mathrm{dt}}=-\frac{{\mathrm{dC}}_{{\mathrm{NO}}_{3}^{-}}}{\mathrm{dt}}-\frac{{\mathrm{dC}}_{{\mathrm{NO}}_{2}^{-}}}{\mathrm{dt}}{=k}_{2}\;C_{{\mathrm{NO}}_{3}^{-}}{+k}_{5}\;C_{{\mathrm{NO}}_{2}^{-}}$$ (14)

$$\frac{{\mathrm{dC}}_{{\mathrm{NH}}_{4}^{+}}}{\mathrm{dt}}=-\frac{{\mathrm{dC}}_{{\mathrm{NO}}_{3}^{-}}}{\mathrm{dt}}-\frac{{\mathrm{dC}}_{{\mathrm{NO}}_{2}^{-}}}{\mathrm{dt}}=k_{3}\;C_{{\mathrm{NO}}_{3}^{-}}+k_{4}\;C_{{\mathrm{NO}}_{2}^{-}}$$ (15)

Where k_1_, k_2_, and k_3_ are the rate constants for reduction of NO_3_^-^ to NO_2_^-^, N_2_ and NH_4_^+^, respectively; k_4_ and k_5_ are the rate constants for reduction of NO_2_^-^ to NH_4_^+^ and N_2_.

**Table 7:** First-order kinetics of catalytic denitrification with different catalysts.

Catalysts	Kinetic equation	R^2^	Rate constant 10^2^ (min^-1^)					
			k	k_1_	k_2_	k_3_	k_4_	k_5_
**Pd-Ag/SiO**_**2**_	y = 0.0077x+0.9763	0.9972	0.77	0.14	0.43	0.26	0.37	0.53
**Pd-Ag/diatomite**	y = 0.006x+0.9939	0.9976	0.60	0.08	0.32	0.24	0.32	0.42
**Pd-Ag/kaolin**	y = 0.0121x+1.0223	0.9968	1.21	0.23	0.79	0.35	0.47	0.86
**Pd-Ag/γ-Al**_**2**_ **O**_**3**_	y = 0.0209x+0.8919	0.9977	2.09	0.46	1.12	0.68	0.81	1.24
**Pd-Ag/silica gel**	y = 0.0094x+0.9799	0.9964	0. 94	0.15	0.61	0.29	0.43	0.73
**Pd-Ag/graphene**	y = 0.0414x+0.5349	0.9982	4.14	0.88	2.11	1.21	1.32	2.25

Results indicated that different catalysts performed distinct reaction rates in catalytic denitrification, which can be explained by k value in Table 7. According to the calculation, for each catalytic process, the summation of k_1,_ k_2_, k_3_ that stands for the overall reaction rate constant was close to k, which implies the catalytic process is a stepwise process. Results indicated that compared to other catalysts, Pd-Ag/graphene showed a higher catalytic rate, which has been proved by data in Table 2. This may be due to the unique properties of graphene, including the porous structure, active surface area, outstanding electronic properties and promising mechanical and thermal stability [33].

## Conclusion

Response surface methodology was used to optimize parameters of catalytic reduction of nitrate. Results indicated that the application of response surface methodology was proved to be feasible. 71.6% of N_2_ Selectivity was obtained under the optimum conditions: 5.1 pH, 127 min reaction time, 3.2 mass ration (Pd: Ag), and 4.2 g/L Fe^0^. However, the cations (K^+^, Na^+^, Ca^2+^, Mg^2+^, and Al^3+^) and anions (Cl^-^, SO_4_^2-^, and HCO_3_^-^) in water body performed different influence on catalytic denitrification. Study on reaction mechanism found that the catalytic denitrification can be achieved with deoxidization processes. Additionally, as the components of catalyst, active ingredients (Pd-Ag) and carrier (graphene) played different role in the catalytic denitrification. The catalytic process could be better explained by first order kinetic model.
